# Exploring the role of admiration in mitigating athlete burnout: an emotional perspective

**DOI:** 10.3389/fpsyg.2026.1754470

**Published:** 2026-03-06

**Authors:** Weihong Xu, Siyu Tian, Tao Song, Si Chen, Yang Pan

**Affiliations:** 1 School of Physical Education, Wuhan Sports University, Wuhan, Hubei, China; 2 School of Sport Education, Tianjin University of Sport, Tianjin, China; 3 Qingdao Badminton and Swimming Sports Management Center, Qingdao, Shandong, China; 4 School of Nursing and Rehabilitation, Cheeloo College of Medicine, Shandong University, Jinan, China; 5 Department of Physical Education, Tianjin Medical University, Tianjin, China

**Keywords:** admiration, adolescents, affect, athlete burnout, emotion

## Abstract

**Introduction:**

Athlete burnout presents a significant challenge for adolescent athletes, with profound implications for their mental health and career progression. This study employs an emotional perspective to examine the relationships between admiration, positive affect, negative affect, and athlete burnout, aiming to identify effective strategies for mitigating burnout among this population.

**Methods:**

The study utilized the “Admiration Scale”, the “Positive and Negative Affect Schedule” and the “Athlete Burnout Questionnaire” to collect data. A total of 1,544 adolescent athletes participated in the study. The collected data were analyzed using three parallel mediation models within the Process macro of SPSS software to elucidate the relationship between admiration and each dimension of athlete burnout, as well as to examine the mediating roles of positive affect and negative affect in this relationship.

**Results:**

Admiration had a significant negative relationship with athlete burnout. There were significant differences in the reduced sense of achievement and devaluation of sports practice among the adolescent athletes who took different objects as models of admiration. Positive affect and negative affect indirectly associate the relationship between admiration and athlete burnout (physical and emotional exhaustion, reduced sense of achievement, devaluation of sports practice). Furthermore, the mediating effect of positive affect was stronger than that of negative affect in all three models.

**Conclusions:**

Admiration may serve a role in managing symptoms of burnout in the adolescent athlete, and that this effect occurs through the effect on positive affect and negative affect.

## 1 Introduction

Athlete burnout, a distinct psychological symptom observed in athletes, denotes a decline in mental functioning resulting from the prolonged depletion of psychological and physiological resources under various stressors. This condition encompasses three dimensions: physical and emotional exhaustion, reduced sense of achievement and devaluation of sports practice (Raedeke and Smith, 2001). Physical and emotional exhaustion were associated with high-intensity training and competition; reduced sense of achievement denotes an athlete's failure to meet personal goals or a performance level that falls below expectations; devaluation of sports practice encompasses a loss of interest in sports or the development of resentment toward sports (Raedeke, 1997). Quite a few studies have shown that burnout has serious harm to athletes in sports ability and mental health, which will lead to the decline of athletes motivation and sports performance, and even make athletes withdraw from sport (Gustafsson et al., 2017).

Adolescence is an important period of personal physical and mental development (Sawyer et al., 2012). Adolescents undergo significant development in cognitive, psychological, emotional, and social domains, encountering numerous internal and external changes across physical, emotional, and social dimensions (Omrani et al., 2019). Additionally, adolescent athletes experience the compounded pressures of academic and training, and may confront challenges such as early specialization and overtraining (Chiou et al., 2020). The inherent instability of adolescence, coupled with the additional challenges encountered by adolescent athletes, renders this demographic more susceptible to experiencing symptoms of burnout. Severe athlete burnout in adolescence can significantly harm both their sports career and overall life development (Brenner et al., 2019). Therefore, it is exceedingly important to prevent and alleviate the athlete burnout of adolescent athletes and reduce the risk of athlete burnout of adolescent athletes.

Admiration is a high liking and respect for the excellent person or role models, and a positive emotional experience is generated when seeing excellent behavior or quality of others (Chen et al., 2011). The pursuit of the best possible results is a defining characteristic of competitive sports. In the field of competitive sports, people can show their excellent abilities and quality in all aspects, and even accomplish the performance of challenging the limits of humans (Fletcher and Sarkar, 2012). Individuals feel admiration when they see extraordinary skills, talents, or achievements (Algoe and Haidt, 2009). Therefore, people can often find their own role models from various fields of sport, which makes sports provide an opportunity to create a sense of admiration (Smith, 2020). Besides, unlike ordinary personal emotions, admiration is person-centered emotion (Onu et al., 2016). It motivates people to concentrate on their admired figures, seek closeness to them, and strive for greater achievements (Algoe and Haidt, 2009; Henrich and Gil-White, 2001). Establishing these achievement goals constitutes an efficacious strategy for mitigating burnout (Ingrell et al., 2019).

Indeed, in the general concept of public, admiration often brings many psychological and behavioral benefits, with motivational characteristics (Archer, 2019). Through a qualitative study, Meng-Lewis et al. (2021) found that by admiring celebrities who are considered to have similar interests and attributes, people may have positive thoughts about their lives and be more active in seeking new opportunities or engaging in self-growth. Zagzebski (2018) similarly claimed that admiration played a crucial role in education (especially moral education) and personal development. However, some studies have presented different opinions. van der Rijt (2018) put forward the “Vice of Admiration”, arguing that the inability of admirers to engage with the admired on an equal footing can significantly undermine the admirer's self-esteem. Consequently, this sense of admiration may adversely affect the individual's emotional and mental wellbeing. Based on the academic circles about the impact of admiration on individuals is positive or negative debate, as well as the special environment of competitive sport. It is necessary to further explore the psychological value of admiration in competitive sport. The relationship between admiration and athlete burnout remains to be tested.

Numerous researches have also discussed the value and significance of positive affect (e.g., inspired, enthusiastic, attentive) and negative affect (e.g., upset, distressed, ashamed) on athlete burnout. Moen et al. (2019) found that positive and negative affects serve as significant mediators in elucidating the relationship between resilience and athlete burnout, as well as between perceived stress and athlete burnout. Gustafsson et al. (2015) identified positive and negative affects as antecedent factors contributing to burnout symptoms in athletes. In addition, by following athletes throughout the season, Lemyre et al. (2006) found that fluctuations of negative and positive affects significantly predicted athletes' susceptibility to burnout. These studies illustrated the importance of emotion in athlete burnout. Besides, Schindler (2014) notes that admiration has a very special relationship with individual emotions, and that admiration provides a potential source of both positive and negative emotions. Due to the admiration is an evaluative outcome that involves both cognitive and affective aspects and may provide psychological support and even influence an individual's cognitive evaluation process (Smith, 2020). Therefore, it can be considered that admiration will act on the individual's positive affect and negative affect, and thus have a connection with the athlete burnout.

Based on above, this study aims to explore the possible relationship between admiration and the three dimensions of athlete burnout (physical and emotional exhaustion, reduced sense of achievement, devaluation of sports practice) of adolescent athletes. We tested two hypotheses: (1) Admiration is significantly correlated with the physical and emotional exhaustion, reduced sense of achievement, devaluation of sports practice of adolescent athletes; and (2) admiration can affect three main manifestations of athlete burnout: physical and emotional exhaustion, reduced sense of achievement, devaluation of sports practice through the intermediary of positive affect and negative affect.

## 2 Methods

### 2.1 Participants and procedures

This study used cluster sampling to contact the principals and managers of 5 sports schools and sports management centers in Yantai and Qingdao, Shandong Province to recruit participants. The study design was in accordance with the Declaration of Helsinki and approved by the Ethics Committee (No. 2022-R-15). A questionnaire survey was conducted on adolescent athletes during class or the training ground. The examination administrator was responsible for the whole questionnaire filling process and can answer questions. Before completing the questionnaire, the researchers assured the participants that their answers were completely anonymous and would not affect them. The questionnaire took about 45 min to complete approximately. A total of 1,578 questionnaires were distributed, and the effective rate was 97.85%. Data screening found that 34 participants did not fill in completely, so they were excluded. The responses of the remaining 1,544 participants were analyzed. The requisite minimum sample size for this study was calculated to be 1,044 participants utilizing G^*^Power analysis. The calculation was conducted utilizing the statistical test of the “*t*-tests, Correlation: Point biserial model,” with the power analysis type of “*A priori*: Compute required sample size”. This calculation was based on a two-tailed test, with the confidence level set at 0.05. The actual sample size utilized in the study met this requirement.

### 2.2 Questionnaire

#### 2.2.1 Admiration

Admiration priming adopts the way of situational recall, asking subjects to recall relevant situations according to the guidance and describe the recalled situations in detail as much as possible to activate admiration emotion. Among them, the instruction is “Admiration is the emotion and feeling caused by people with excellent ability/virtue. Usually, this excellent ability/virtue can be understood or achieved by individuals. Please describe a person you admire (such as Olympic champion, sports star, coach, teammate, etc.) in terms of ability/virtue in as much detail as possible. And examples of achievements/virtues that you admire, and describe his or her ability traits/virtue qualities or ability performance/virtue behavior that you admire”. After the emotion of admiration was activated, the admiration scale composed of 12 items was used to measure the admiration of adolescent athletes for their admiration objects. The response was measured on the Likert scale with a 5-point scale, “1” for complete non-compliance and “5” for complete compliance (Chen, 2019). Add the scores of these 12 items to calculate the score of the scale. The higher the value, the stronger the admiration. Previous researches have demonstrated that the scale possesses robust reliability and validity within the Chinese context, thereby establishing its efficacy as a tool for assessing personal admiration (Zou et al., 2024; Chen, 2019). The Cronbach's α coefficient of the scale was 0.930.

#### 2.2.2 Positive- negative affect

To measure positive affect and negative affect of adolescent athletes, the Chinese version of Positive and Negative Affect Schedule designed by Watson et al. (1988) and adapted by Huang et al. (2003) was used. The scale consists of 20 items, including two dimensions of positive affect and negative affect. Ten of the items measured positive affect (e.g., Attentive) and 10 items measured negative affect (e.g., Nervous). Each item was rated on a Likert scale ranging from 1 (not at all) to 5 (extremely). The scale has shown good reliability and validity in previous studies on Chinese adolescent (Zhang et al., 2015). Cronbach's alpha coefficient values for the positive affect subscale and negative affect subscale were 0.832 and 0.846 respectively in the present study.

#### 2.2.3 Athlete burnout

The Athlete Burnout Questionnaire (ABQ) developed by Raedeke and Smith was used to measure the degree of athlete burnout of adolescent athletes (Raedeke and Smith, 2001). The scale includes three dimensions: physical and emotional exhaustion, reduced sense of achievement and devaluation of sports practice. Each dimension has 5 items, consisting of 15 items in total. The five-point Likert-type is utilized, 1–5 indicates from “never” to “always”. And the first and 14th items are reverse-coded items. The total score of the ABQ is summed up in the all items. The higher the score, the higher the degree of athlete burnout of adolescent athletes. Previous studies have shown that the scale has a good level of reliability and validity in Chinese samples (Liu et al., 2023). In this study, Cronbach' s α for the total scale was 0.882, for the subscale of physical and emotional exhaustion, reduced sense of achievement, and devaluation of sports practice were 0.806, 0.676, and 0.783 respectively.

### 2.3 Statistical analysis

After input of paper questionnaire data into Excel for verification, all statistical analysis was performed in SPSS 24.0 (IBM, Armonk, NY, USA). Descriptive statistics were used to describe the mean and standard deviation of each variable, and Pearson correlation analysis was used to calculate the correlation between variables, to provide a basis for subsequent analysis. And the One-way ANOVA was used to test the group differences of athlete burnout with different admiration objects. Following, the three models were established, in which the independent variable was admiration, the dependent variable was physical and emotional exhaustion, reduced sense of achievement and devaluation of sports practice respectively, and the mediating variable was positive affect and negative affect. Hayes PROCESS macro (version 3.0) based on regression analysis was used to conduct the mediation analysis on the model (Hayes, 2013), with demographic variables (i.e., sex, age, training years, sports projects, sports grade, and changed sports projects) as covariates. All variables were standardized before entering the mediation model. The direct, indirect, and total effects, as well as differences between indirect effects, were calculated using PROCESS based on 5,000 bootstrap samples, and 95% deviation corrected confidence intervals (CIS) were estimated. CI does not contain zero, indicating that the effect is considered significant. The significance level was set to α = 0.05.

## 3 Results

### 3.1 Demographics

The participants of this study included 1,544 adolescent athletes. Among them, there were 852 male adolescent athletes and 692 female adolescent athletes, with an average age of 14.43 (SD = 1.92). Adolescent athletes' average training of 3.03 (SD = 1.86) years. Involving 22 sports, including track and field, swimming, football, basketball, volleyball, rugby, weightlifting, judo, rock climbing and shooting, and so on. There are 12 master sportsman athletes, 138 first-level athletes, 366 second-level athletes, and 1,028 non-level athletes according to the criteria of the Chinese State General Administration of Sports (https://www.sport.gov.cn/jts/n5005/c351208/part/211968.pdf). Among them, 640 adolescent athletes have changed their sports projects.

### 3.2 Common method bias testing

Harman's single-factor test was applied to estimate the common method bias. After analysis, 7 eigenvalues greater than 1 were extracted. The first factor to explain the variance was 22.453%, which was less than the 40% required by critical standards, demonstrating that the questionnaires used in the current study had no significant issue with common method biases (Tang and Wen, 2020).

### 3.3 The association of admiration object with athlete burnout

In the study, the admiration objects of adolescent athletes were divided into eight categories: sports stars of their own related events, sports stars of other events, scientists, teammates, coaches, family members, teachers, and others. The results of one-way ANOVA test showed that except for the physical and emotional exhaustion of athlete burnout (*F* = 1.202, *p* = 0.298), athletes with different admiration objects had obvious group differences in reduced sense of achievement (*F* = 2.917, *p* = 0.005) and devaluation of sports practice (*F* = 2.698, *p* = 0.009). *Post hoc* multiple analysis of the dimensions of reduced sense of achievement and devaluation of sports practice can be found that in terms of reduced sense of achievement, athletes who admire coaches were often the least likely to feel the reduction of the sense of achievement. In the case of devaluation of sports practice, athletes who admire sports stars in their own related projects often had the least sports negative evaluation. See Table 1 for details.

**Table 1:** Results of *post hoc* multiple comparisons of the relationship between object of admiration and athlete burnout.

**Dependent variable**	**(I) Object of admiration**	**(J) Object of admiration**	**Mean difference (I-J)**	***SD***	***P***	95% confidence interval	
						**Lower limit**	**Upper limit**
Reduced sense of achievement	Sports stars of their own related events	Sports stars of other events	−0.149	0.326	0.647	−0.790	0.490
		Scientists	−0.116	0.840	0.890	−1.760	1.530
		Teammates	−0.890^*^	0.306	0.004	−1.490	−0.290
		Coaches	0.050	0.250	0.843	−0.440	0.540
		Family members	−1.043^*^	0.489	0.033	−2.000	−0.080
		Teachers	−0.944^*^	0.375	0.012	−1.680	−0.210
		Others	−0.554	0.820	0.500	−2.160	1.060
	Sports stars of other events	Sports stars of their own related events	0.149	0.326	0.647	−0.490	0.790
		Scientists	0.033	0.859	0.969	−1.650	1.720
		Teammates	−0.741^*^	0.356	0.037	−1.440	−0.040
		Coaches	0.199	0.309	0.520	−0.410	0.800
		Family members	−0.894	0.522	0.087	−1.920	0.130
		Teachers	−0.795	0.416	0.056	−1.610	0.020
-		Others	−0.405	0.840	0.630	−2.050	1.240
	Scientists	Sports stars of their own related events	0.116	0.840	0.890	−1.530	1.760
		Sports stars of other events	−0.033	0.859	0.969	−1.720	1.650
		Teammates	−0.774	0.851	0.363	−2.440	0.900
		Coaches	0.166	0.833	0.842	−1.470	1.800
		Family members	−0.927	0.933	0.320	−2.760	0.900
		Teachers	−0.828	0.878	0.346	−2.550	0.900
		Others	−0.438	1.142	0.701	−2.680	1.800
	Teammates	Sports stars of their own related events	0.890^*^	0.306	0.004	0.290	1.490
		Sports stars of other events	0.741^*^	0.356	0.037	0.040	1.440
		Scientists	0.774	0.851	0.363	−0.900	2.440
		Coaches	0.940^*^	0.288	0.001	0.380	1.500
		Family members	−0.153	0.509	0.764	−1.150	0.850
		Teachers	−0.053	0.401	0.894	−0.840	0.730
		Others	0.336	0.833	0.686	−1.300	1.970
	Coaches	Sports stars of their own related events	−0.050	0.250	0.843	−0.540	0.440
		Sports stars of other events	−0.199	0.309	0.520	−0.800	0.410
		Scientists	−0.166	0.833	0.842	−1.800	1.470
		Teammates	−0.940^*^	0.288	0.001	−1.500	−0.380
		Family members	−1.093^*^	0.478	0.022	−2.030	−0.160
		Teachers	−0.993^*^	0.360	0.006	−1.700	−0.290
		Others	−0.604	0.814	0.458	−2.200	0.990
	Family members	Sports stars of their own related events	1.043^*^	0.489	0.033	0.080	2.000
		Sports stars of other events	0.894	0.522	0.087	−0.130	1.920
		Scientists	0.927	0.933	0.320	−0.900	2.760
		Teammates	0.153	0.509	0.764	−0.850	1.150
		Coaches	1.093^*^	0.478	0.022	0.160	2.030
		Teachers	0.099	0.553	0.857	−0.990	1.180
		Others	0.489	0.916	0.593	−1.310	2.290
	Teachers	Sports stars of their own related events	0.944^*^	0.375	0.012	0.210	1.680
						**Lower limit**	**Upper limit**
		Sports stars of other events	0.795	0.416	0.056	−0.020	1.610
		Scientists	0.828	0.878	0.346	−0.900	2.550
		Teammates	0.053	0.401	0.894	−0.730	0.840
		Coaches	0.993^*^	0.360	0.006	0.290	1.700
		Family members	−0.099	0.553	0.857	−1.180	0.990
		Others	0.390	0.860	0.650	−1.300	2.080
	Others	Sports stars of their own related events	0.554	0.820	0.500	−1.060	2.160
		Sports stars of other events	0.405	0.840	0.630	−1.240	2.050
		Scientists	0.438	1.142	0.701	−1.800	2.680
		Teammates	−0.336	0.833	0.686	−1.970	1.300
		Coaches	0.604	0.814	0.458	−0.990	2.200
		Family members	−0.489	0.916	0.593	−2.290	1.310
		Teachers	−0.390	0.860	0.650	−2.080	1.300
Devaluation of sports practice	Sports stars of their own related events	Sports stars of other events	−0.598	0.325	0.066	−1.240	0.040
		Scientists	−0.277	0.838	0.741	−1.920	1.370
		Teammates	−0.835^*^	0.305	0.006	−1.430	−0.240
		Coaches	−0.156	0.250	0.533	−0.650	0.330
		Family members	−1.372^*^	0.488	0.005	−2.330	−0.420
		Teachers	−0.912^*^	0.374	0.015	−1.650	−0.180
		Others	−1.208	0.819	0.140	−2.810	0.400
	Sports stars of other events	Sports stars of their own related events	0.598	0.325	0.066	−0.040	1.240
		Scientists	0.321	0.857	0.708	−1.360	2.000
		Teammates	−0.238	0.355	0.503	−0.930	0.460
		Coaches	0.442	0.308	0.152	−0.160	1.050
		Family members	−0.775	0.521	0.137	−1.800	0.250
		Teachers	−0.314	0.415	0.449	−1.130	0.500
		Others	−0.610	0.838	0.467	−2.250	1.030
	Scientists	Sports stars of their own related events	0.277	0.838	0.741	−1.370	1.920
		Sports stars of other events	−0.321	0.857	0.708	−2.000	1.360
		Teammates	−0.559	0.850	0.511	−2.230	1.110
		Coaches	0.121	0.831	0.884	−1.510	1.750
		Family members	−1.095	0.931	0.240	−2.920	0.730
		Teachers	−0.635	0.877	0.469	−2.350	1.080
		Others	−0.931	1.140	0.414	−3.170	1.300
	Teammates	Sports stars of their own related events	0.835^*^	0.305	0.006	0.240	1.430
		Sports stars of other events	0.238	0.355	0.503	−0.460	0.930
		Scientists	0.559	0.850	0.511	−1.110	2.230
		Coaches	0.680^*^	0.287	0.018	0.120	1.240
		Family members	−0.537	0.508	0.291	−1.530	0.460
		Teachers	−0.077	0.400	0.848	−0.860	0.710
		Others	−0.372	0.831	0.654	−2.000	1.260
	Coaches	Sports stars of their own related events	0.156	0.250	0.533	−0.330	0.650
		Sports stars of other events	−0.442	0.308	0.152	−1.050	0.160
		Scientists	−0.121	0.831	0.884	−1.750	1.510
		Teammates	−0.680^*^	0.287	0.018	−1.240	−0.120
		Family members	−1.217^*^	0.477	0.011	−2.150	−0.280
		Teachers	−0.756^*^	0.359	0.035	−1.460	−0.050
		Others	−1.052	0.812	0.195	−2.640	0.540
	Family members	Sports stars of their own related events	1.372^*^	0.488	0.005	0.420	2.330
		Sports stars of other events	0.775	0.521	0.137	−0.250	1.800
		Scientists	1.095	0.931	0.240	−0.730	2.920
		Teammates	0.537	0.508	0.291	−0.460	1.530
		Coaches	1.217^*^	0.477	0.011	0.280	2.150
		Teachers	0.460	0.552	0.405	−0.620	1.540
		Others	0.165	0.914	0.857	−1.630	1.960
	Teachers	Sports stars of their own related events	0.912^*^	0.374	0.015	0.180	1.650
		Sports stars of other events	0.314	0.415	0.449	−0.500	1.130
		Scientists	0.635	0.877	0.469	−1.080	2.350
		Teammates	0.077	0.400	0.848	−0.710	0.860
		Coaches	0.756^*^	0.359	0.035	0.050	1.460
		Family members	−0.460	0.552	0.405	−1.540	0.620
		Others	−0.296	0.858	0.731	−1.980	1.390
	Others	Sports stars of their own related events	1.208	0.819	0.140	−0.400	2.810
		Sports stars of other events	0.610	0.838	0.467	−1.030	2.250
		Scientists	0.931	1.140	0.414	−1.300	3.170
		Teammates	0.372	0.831	0.654	−1.260	2.000
		Coaches	1.052	0.812	0.195	−0.540	2.640
		Family members	−0.165	0.914	0.857	−1.960	1.630
		Teachers	0.296	0.858	0.731	−1.390	1.980

^*^ *p* < 0.05.

### 3.4 Correlation analysis

The Means, standard deviations, and a correlation matrix for admiration, positive affect, negative affect, and athlete burnout were presented in Table 2. A bivariate correlation analysis showed that all the variables were significantly correlated (*Ps* < 0.01). To be specific, admiration was positively correlated with positive affect and negatively correlated with negative affect and athlete burnout (physical and emotional exhaustion, reduced sense of achievement, devaluation of sports practice); positive affect was negatively correlated with negative affect and athlete burnout (physical and emotional exhaustion, reduced sense of achievement, devaluation of sports practice); negative affect was positively correlated with burnout (physical and emotional exhaustion, reduced sense of achievement, devaluation of sports practice).

**Table 2:** Descriptive statistics and correlation matrix for admiration, positive affect, negative affect, and athlete burnout in adolescent athletes.

**Variable**	**1**	**2**	**3**	**4**	**5**	**6**	**7**
1. Admiration	—						
2. Positive affect	0.334^**^	—					
3. Negative affect	−0.111^**^	−0.194^**^	—				
4. Physical and emotional exhaustion	−0.127^**^	−0.324^**^	0.489^**^	—			
5. Reduced sense of achievement	−0.227^**^	−0.472^**^	0.405^**^	0.575^**^	—		
6. Devaluation of sports practice	−0.178^**^	−0.324^**^	0.379^**^	0.606^**^	0.580^**^	—	
7. Athlete burnout	−0.205^**^	−0.436^**^	0.502^**^	0.868^**^	0.836^**^	0.849^**^	—
*M*	46.431	32.799	21.696	11.749	12.954	9.582	33.285
*SD*	8.524	6.988	6.821	4.202	3.671	3.662	9.825

M, mean; SD, standard deviation; ^**^p < 0.01.

### 3.5 The regression analysis

Table 3 demonstrated the regression coefficients of the mediation model with admiration as the independent variable, physical and emotional exhaustion as the dependent variable, positive affect and negative affect as the mediating variables, and demographic variables (i.e., sex, age, training years, sports projects, sports grade, and changed sports projects) as the covariables. When no mediating variables were added, the regression coefficient of admiration on physical and emotional exhaustion was significant (β = −0.139, *p* < 0.001). After adding the mediation variable, although the regression coefficient between admiration and physical and emotional exhaustion was not statistically significant (β = −0.001, *p* > 0.05), the regression coefficient of admiration with positive affect (β = 0.349, *p* < 0.001) and negative affect (β = −0.122, *p* < 0.001) were significant. Meanwhile, the regression coefficients for positive affect on physical and emotional exhaustion (β = −0.243, *p* < 0.001) and negative affect on physical and emotional exhaustion (β = 0.441, *p* < 0.001) were also significant.

**Table 3:** Regression coefficients of the mediating of positive affect and negative affect between admiration and physical and emotional exhaustion.

**Outcome variables**	**Predictive variables**	Goodness-of-fit indices			Regression coefficient and significance	
		***R***	***R** ^2^*	***F***	β	***t***
Physical and emotional exhaustion		0.239	0.057	13.298^***^		
	Admiration				−0.139	−5.582^***^
Positive affect		0.417	0.176	46.067^***^		
	Admiration				0.349	14.943^***^
Negative affect		0.227	0.052	11.936^***^		
	Admiration				−0.122	−4.862^***^
Physical and emotional exhaustion		0.562	0.316	78.697^***^		
	Admiration				−0.001	−0.027
	Positive affect				−0.243	−10.386^***^
	Negative affect				0.441	20.179^***^

^***^ *p* < 0.001; Demographic variables as covariances.

Table 4 showed the regression coefficients of the mediation model with admiration as the independent variable, reduced sense of achievement as the dependent variable, positive affect and negative affect as the mediating variables, and demographic variables as the covariables. Firstly, the regression coefficient of admiration on reduced sense of achievement was significant (β = −0.237, *p* < 0.001). Secondly, after adding the mediation variable, the regression coefficient between admiration and reduced sense of achievement was statistically significant (β = −0.065, *p* < 0.01). In addition, the regression coefficient of admiration with positive affect (β = 0.349, p < 0.001) and negative affect (β = −0.122, *p* < 0.001) were significant. Meanwhile, the regression coefficients for positive affect on reduced sense of achievement (β = −0.380, *p* < 0.001) and negative affect on reduced sense of achievement (β = 0.315, *p* < 0.001) were also significant.

**Table 4:** Regression coefficients of the mediating of positive affect and negative affect between admiration and reduced sense of achievement.

**Outcome variables**	**Predictive variables**	Goodness-of-fit indices			Regression coefficient and significance	
		***R***	***R** ^2^*	***F***	β	***t***
Reduced sense of achievement		0.310	0.096	23.349^***^		
	Admiration				−0.237	−9.679^***^
Positive affect		0.417	0.176	46.067^***^		
	Admiration				0.349	14.943^***^
Negative affect		0.227	0.052	11.936^***^		
	Admiration				−0.122	−4.863^***^
Reduced sense of achievement		0.581	0.338	86.992^***^		
	Admiration				−0.065	−2.909^**^
	Positive affect				−0.380	−16.503^***^
	Negative affect				0.315	14.649^***^

^**^ *p* < 0.01; ^***^ *p* < 0.001; Demographic variables as covariances.

Table 5 indicated the regression coefficients of the mediation model with admiration as the independent variable, devaluation of sports practice as the dependent variable, positive affect and negative affect as the mediating variables, and demographic variables as the covariables. The regression coefficient of admiration on devaluation of sports practice was significant when no mediating variables were added (β = −0.217, *p* < 0.001). As well, after adding mediation variables, the regression coefficient between admiration and devaluation of sports practice was statistically significant (β = −0.060, *p* < 0.05). Besides, the regression coefficient of admiration with positive affect (β = 0.349, *p* < 0.001) and negative affect (β = −0.122, *p* < 0.001) were significant. Simultaneously, the regression coefficients for positive affect on devaluation of sports practice (β= −0.241, *p* < 0.001) and negative affect on devaluation of sports practice (β = 0.317, *p* < 0.001) were also significant.

**Table 5:** Regression coefficients of the mediating of positive affect and negative affect between admiration and devaluation of sports practice.

**Outcome variables**	**Predictive variables**	Goodness-of-fit indices			Regression coefficient and significance	
		***R***	***R** ^2^*	***F***	β	***t***
Devaluation of sports practice		0.241	0.058	13.537^***^		
	Admiration				−0.217	−8.533^***^
Positive affect		0.417	0.174	46.067^***^		
	Admiration				0.349	14.943^***^
Negative affect		0.227	0.052	11.936		
	Admiration				−0.122	−4.862^***^
Devaluation of sports practice		0.471	0.222	48.526^***^		
	Admiration				−0.060	−2.473^*^
	Positive affect				−0.241	−9.636^***^
	Negative affect				0.317	13.601^***^

^*^ *p* < 0.05; ^***^ *p* < 0.001; Demographic variables as covariances.

**Figure 1:**
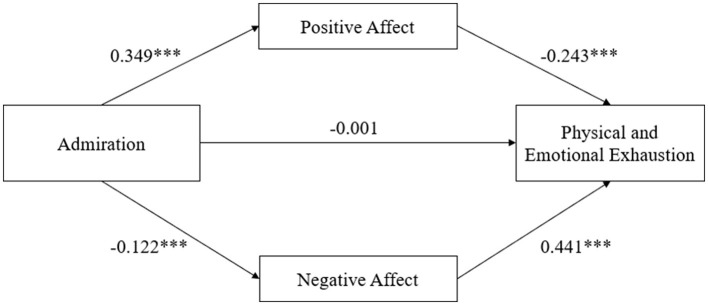
Parallel mediation model of positive affect and negative affect between admiration and physical and emotional exhaustion; ^***^p < 0.001; Demographic variables as covariances.

**Figure 2:**
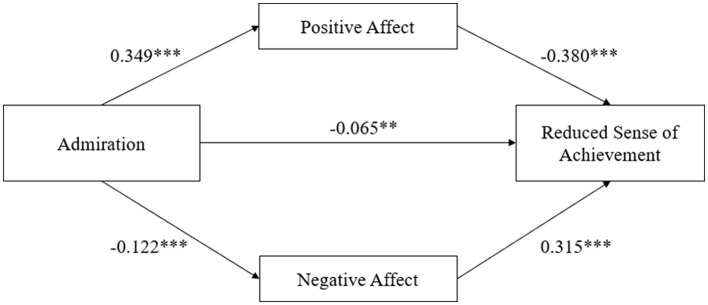
Parallel mediation model of positive affect and negative affect between admiration and reduced sense of achievement; ^**^p < 0.01; ^***^p < 0.001; Demographic variables as covariances.

**Figure 3:**
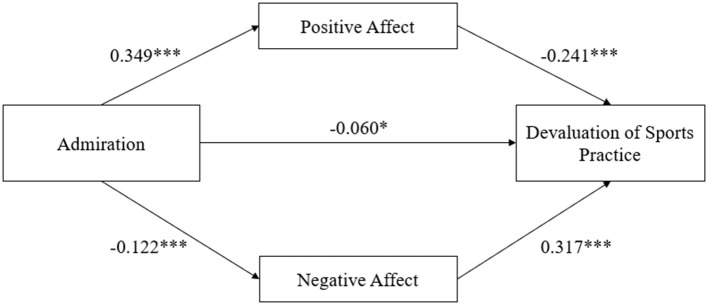
Parallel mediation model of positive affect and negative affect between admiration and devaluation of sports practice; ^*^p < 0.05; ^***^p < 0.001; Demographic variables as covariances.

These results illustrated that positive affect and negative affect completely mediated the relationship between admiration with physical and emotional exhaustion, and partially mediated the relationship between admiration and reduced sense of achievement, and devaluation of sports practice. The regression weights of the path analyses are shown in Figures 1–3.

### 3.6 Mediation analysis

Mediation analysis based on 5,000 bootstrap samples was conducted to estimate the indirect effects of admiration on athlete burnout (physical and emotional exhaustion, reduced sense of achievement, devaluation of sports practice) mediated by positive affect and negative affect.

As shown in the Table 6, in the model of physical and emotional exhaustion, the direct effect was not significant (effect value = −0.001, 95% CI:−0.045, 0.044), but the total indirect effect was significant (effect value = −0.139, 95% CI:−0.173,−0.107). The indirect effect of positive affect (effect value = −0.085, 95% CI:−0.107,−0.064), and negative affect (effect value = −0.054, 95% CI:−0.078,−0.031) were all significant. In addition, there was a significant difference between the effect sizes of the two mediators (effect value = −0.031, 95% CI:−0.062,−0.001).

**Table 6:** Mediating effects of positive affect and negative affect between admiration and physical and emotional exhaustion.

**Effect type**	**Effect**	**Bootstrap *SE***	**Bootstrap 95% CI lower limit**	**Boot 95% CI upper limit**
Total effect	−0.139	0.025	−0.188	−0.090
Direct effect	−0.001	0.023	−0.045	0.044
Indirect effect	−0.139	0.017	−0.173	−0.107
A → PA → PEE	−0.085	0.109	−0.107	−0.064
A → NA → PEE	−0.054	0.012	−0.078	−0.031
C1	−0.031	0.016	−0.062	−0.001

A, admiration; PA, positive affect; NA, negative affect; PEE, physical and emotional exhaustion; C1 = SA → PA → PEE-SA → NA → PEE; Demographic variables as covariances.

As illustrated in the Table 7, in the model of reduced sense of achievement, the direct effect (effect value = −0.065, 95% CI: −0.109,−0.021) and the total indirect effect were all significant (effect value = −0.171, 95% CI:−0.205, −0.141). The indirect effect of positive affect (effect value = −0.133, 95% CI: −0.159,−0.109), and negative affect (effect value = −0.038, 95% CI: −0.057, −0.022) were all significant. Moreover, there was a significant difference between the effect sizes of the two mediators (effect value = −0.095, 95% CI: −0.124, −0.066).

**Table 7:** Mediating effects of positive affect and negative affect between admiration and reduced sense of achievement.

**Effect type**	**Effect**	**Bootstrap *SE***	**Bootstrap 95% CI lower limit**	**Boot 95% CI upper limit**
Total effect	−0.237	0.025	−0.285	−0.189
Direct effect	−0.065	0.023	−0.109	−0.021
Indirect effect	−0.171	0.017	−0.205	−0.141
A → PA → RSA	−0.133	0.013	−0.159	−0.109
A → NA → RSA	−0.038	0.009	−0.057	−0.022
C1	−0.095	0.015	−0.124	−0.066

A, admiration; PA, positive affect; NA, negative affect; RSA, reduced sense of achievement; C1 = SA → PA → RSA- SA → NA → RSA; Demographic variables as covariances.

As shown in the Table 8, in the model of devaluation of sports practice, the direct effect (effect value = −0.060, 95% CI:−0.108,−0.013) and the total indirect effect were all significant (effect value = −0.123, 95% CI: −0.155, −0.095). The indirect effect of positive affect (effect value = −0.084, 95% CI: −0.108, −0.062), and negative affect (effect value = −0.039, 95% CI: −0.058, −0.022) were all significant. Additionally, there was a significant difference between the effect sizes of the two mediators (effect value = −0.046, 95% CI: −0.074, −0.017).

**Table 8:** Mediating effects of positive affect and negative affect between admiration and devaluation of sports practice by process.

**Effect type**	**Effect**	**Bootstrap *SE***	**Bootstrap 95% CI lower limit**	**Boot 95% CI upper limit**
Total effect	−0.183	0.025	−0.232	−0.134
Direct effect	−0.060	0.024	−0.108	−0.013
Indirect effect	−0.123	0.015	−0.155	−0.095
A → PA → DSP	−0.084	0.012	−0.108	−0.062
A → NA → DSP	−0.039	0.009	−0.058	−0.022
C1	−0.046	0.015	−0.074	−0.017

A, admiration; PA, positive affect; NA, negative affect; DSP, devaluation of sports practice; C1 = SA → PA → DSP- SA → NA → DSP; Demographic variables as covariances.

## 4 Discussion

We found that there was a significant negative correlation between admiration and the three dimensions of athlete burnout. This is consistent with hypothesis 1, indicating that adolescent athletes with higher feelings of admiration are less likely to exhibit three symptoms of athlete burnout: physical and emotional exhaustion, reduced sense of achievement, devaluation of sports practice. To the best of our knowledge, we provide the first published s quantitative study to examine the positive impact of admiration on athletes, which makes our findings difficult to compare with previous literature. However, since admiration is characterized by motivation (Archer, 2019), the role of motivation in athlete burnout has been repeatedly verified (Cresswell and Eklund, 2005; Gustafsson et al., 2017; Lonsdale et al., 2009). Therefore, motivation, especially autonomous motivation, may also be a valid explanation for the association between admiration and athlete burnout. Admiration enables adolescent athletes to expand themselves by imitating others who are admired and encourages them to strive for their own success (Onu et al., 2016; Schindler et al., 2015). In order to achieve success, this internal driving force makes them obtain powerful psychological resources to resist the emergence of athlete burnout. Furthermore, the work of Immordino-Yang and Sylvan (2010) pointed out that the motivating power of emotions like admiration comes not only from consciously evoking knowledge relevant to one's own situation, but also from unconscious processes related to mind-body interdependence. Therefore, it may be considered that admiration not only affects the psychological state of adolescent athletes at the cognitive level, but also prevents the symptoms of burnout of adolescent athletes by affecting the physiological system that regulates heart rate, blood pressure, and hormones.

Our study found that while different objects of admiration don't cause the physical and emotional exhaustion in adolescent athletes, they do lead to group differences in the reduced sense of achievement and the devaluation of sports practice. Adolescent athletes who admire coaches are often the least likely to feel the reduction of their sense of achievement. Similarly, those who admire sports stars related to their competitive events often receive the least negative sports evaluation. This is because having a sense of admiration for coaches often shows the trust of adolescent athletes in coaches. They will firmly believe that they will eventually achieve excellent results under the guidance of coaches. In this case, the admiration for the coach may act on the sense of achievement of adolescent athletes in a special way of coach social support to reduce symptoms of reduced sense of achievement. On the other hand, coaches who are admired by athletes tend to have a closer relationship with athletes, and their coaching style tends to be more self-supporting. This approach can enhance adolescent athletes' sense of accomplishment in competitive sports and alleviate symptoms of burnout (Choi et al., 2020; Ruser et al., 2021). While, the admiration of sports stars related to their competitive events may make adolescent athletes pay more attention to the advantages and positive effects brought by sports competition. At the same time, they will also focus on the advantages of sports stars, so as to reduce the possibility of negative evaluation of sports.

The results of this study also confirmed hypothesis 2 that positive and negative affects play a parallel mediating role between the admiration and the three main symptoms of athlete burnout. This is inconsistent with van der Rijt's (2018) view that admiration will have a negative impact on individual psychological health, and supports the previous view that admiration will bring people good emotional experiences (Chen et al., 2011). The findings point out that the psychological benefits of admiration are multifaceted, as it is not only positively correlated with the positive affect of adolescent athletes, but also negatively correlated with their negative affect. Moreover, through the role of affect, admiration has a significant impact on the three main symptoms of athlete burnout, which can comprehensively affect the burnout of adolescent athletes.

Specifically, positive and negative affects play a complete mediating role in the model of the relationship between admiration and physical and emotional exhaustion. This suggests that affective responses constitute a significant mechanism through which admiration influences symptoms of physical and emotional exhaustion. Algoe and Haidt (2009) have discussed the physiological and psychological response of admiration in their research, pointing out that when admiration makes people feel pleasant, it can not only trigger physical feelings such as ‘Warm' chest, high energy and increased heart rate, but also inspire and motivate people to work harder for their goals and projects. Physical and emotional exhaustion is the symptom of athlete burnout most closely related to psychological stress and physiological load (Gustafsson et al., 2011). Perhaps it is precise because the emotional response caused by admiration can act on the symptoms of physical and emotional exhaustion through both psychophysiological aspects. Therefore, in the model of physical and emotional exhaustion, positive affect and negative affect show a complete intermediary role.

In the model of the reduced sense of achievement and devaluation of sports practice, positive affect and negative affect played a partial mediating role. Since adolescent athletes with high admiration are more likely to target their role models, they can get a sense of achievement by approaching their excellent behaviors and characters in all aspects (Algoe and Haidt, 2009). Therefore, they will not limit self-growth and self-recognition to the fluctuation of sports performance. In this case, it is easier for adolescent athletes to obtain a variety of positive affect responses in life and training, and avoid being immersed in the negative affect that may be brought about by poor sports performance. This, in turn, reduces the possibility of the symptoms of reduced sense of achievement and devaluation of sports practice. However, some mediating results of the two models suggest that other important factors, possibly variables such as motivation, may play an important role in the relationship between admiration and the two symptoms, so further research is needed.

Additionally, the three models established by the study all show that the role of positive affect is significantly stronger than the role of negative affect in the relationship between admiration and athlete burnout. On the one hand, this result supports the previous view that not only negative affect has an impact on athlete burnout, but also positive affect can play a role in athlete burnout (Gustafsson et al., 2015; Lemyre et al., 2006). On the other hand, it also shows that influence of admiration on positive affect may play a more crucial role in preventing burnout among adolescent athletes compared to its impact on negative affect.

This study has significant implications. Firstly, as far as the author knows, this study is the first time to put forward the mitigation and prevention of athlete burnout from the perspective of admiration, and the quantitative research on the relationship between admiration and burnout of adolescent athletes, which provides a new thought for the research of athlete burnout. Secondly, the findings of this study prove that setting an example for the adolescent athlete and stimulating their admiration is an effective way to manage their burnout symptoms, which helps managers and coaches customize corresponding prevention programs and measures. Finally, this study reveals that positive affect and negative affect parallelly mediate the influence of admiration on athlete burnout, which deeply expounds on the specific psychological mechanism of admiration on burnout of adolescent athletes.

The research still had several limitations. Due to the cross-sectional nature of our survey, although we used situational recall to initiate the admiration of the subjects, we still could not infer the causal relationship from the statistical analysis. Therefore, longitudinal follow-up studies or experimental intervention studies should be conducted in the future to verify the relationship between admiration, positive affect, negative affect, and athlete burnout. Moreover, in this study, the Cronbach's α coefficient for the “reduced sense of achievement” subscale of the Athlete Burnout Questionnaire (ABQ) was determined to be 0.676, which is marginally below the commonly accepted threshold of 0.7. This may primarily stem from the inclusion of two reverse-scored items within this sub-scale, which necessitate participants to possess certain levels of reading comprehension and cognitive transformation abilities. The average age of participants in this study was 14.43 years (± 1.92), and it included some athletes as young as 12 years, whose reading comprehension skills may still be in development, potentially leading to misunderstandings of the questions posed. The age characteristics of the participants, combined with the specific nature of the reverse-scored items, may collectively led to the slightly lower reliability observed in the “reduced sense of achievement” sub-scale. This also underscores the importance of careful selection and adjustment of measurement tools in future research, particularly when assessing younger athlete populations. Additionally, our study was conducted among adolescent athletes, so the results of this study may not be easily generalized to athletes of all ages. For example, if elite athletes are taken as the research object, the results may be inconsistent. Elite athletes are often perceived as figures worthy of admiration, and their experience of admiration, along with their psychological functioning, may differ significantly from that of younger athletes. Further research is required to explore, compare, and validate whether the admiration experienced by elite athletes can be leveraged to manage burnout. Additionally, it is necessary to determine whether the mechanisms underlying admiration in elite athletes align with those in younger athletes. Such investigations will help elucidate the psychological pathways and distinctions in the experience of admiration among athletes at varying levels of competition. The exclusive use of samples from China may restrict the cultural generalizability of the findings. Admiration, as an emotion characterized by unique social construction attributes, may have its modes of expression, eliciting conditions, and psychological functions significantly shaped by cultural values. Future research should be conducted within a multicultural framework to examine the differences in the role of admiration in managing athlete burnout across collectivist and individualist cultures. This approach would offer a theoretical foundation and empirical support for developing culturally adaptive psychological intervention strategies.

## 5 Conclusion

Our study suggests that admiration may serve a role in managing symptoms of burnout in the adolescent athlete. Moreover, high admiration for coaches is more conducive to avoiding the symptoms of a reduced sense of achievement, while athletes who admire sports stars related to their own sports are less likely to show devaluation of sports practice. Our results also demonstrated that both positive affect and negative affect played parallel mediating roles in the relationship between admiration and athlete burnout. It is a feasible method to protect adolescent athletes from burnout by enhancing their sense of admiration to make them feel more positive affect and less negative affect.

## Data Availability

The original contributions presented in the study are included in the article/supplementary material, further inquiries can be directed to the corresponding author.
